# Analysis of serum inflammatory mediators in type 2 diabetic patients and their influence on renal function

**DOI:** 10.1371/journal.pone.0229765

**Published:** 2020-03-04

**Authors:** Liliane Silvano Araújo, Marcos Vinícius da Silva, Crislaine Aparecida da Silva, Maria de Fátima Borges, Heloísa Marcelina da Cunha Palhares, Laura Penna Rocha, Rosana Rosa Miranda Corrêa, Virmondes Rodrigues Júnior, Marlene Antônia dos Reis, Juliana Reis Machado

**Affiliations:** 1 Discipline of General Pathology, Institute of Biological and Natural Sciences of Federal University of Triângulo Mineiro, Uberaba, Minas Gerais, Brazil; 2 Department of Microbiology, Immunology and Parasitology, Institute of Biological and Natural Sciences of Federal University of Triângulo Mineiro, Uberaba, Minas Gerais, Brazil; 3 Discipline of Endocrinology and Metabolism, Health Sciences Institute of Federal University of Triângulo Mineiro, Uberaba, Minas Gerais, Brazil; National Institutes of Health, UNITED STATES

## Abstract

**Aim:**

To evaluate the serum concentrations of inflammatory mediators in patients with type 2 diabetes mellitus (T2DM) with or without renal alteration (RA) function.

**Methods:**

Serum samples from 76 patients with T2DM and 24 healthy individuals were selected. Patients with T2DM were divided into two groups according to eGFR (> or < 60mL/min/1.73m^2^). Cytokines, chemokines and adipokines levels were evaluated using the Multiplex immunoassay and ELISA.

**Results:**

TNFR1 and leptin were higher in the T2DM group with RA than in the T2DM group without RA and control group. All patients with T2DM showed increased resistin, IL-8, and MIP-1α compared to the control group. Adiponectin were higher and IL-4 decreased in the T2DM group with RA compared to the control group. eGFR positively correlated with IL-4 and negatively with TNFR1, TNFR2, and leptin in patients with T2DM. In the T2DM group with RA, eGFR was negatively correlated with TNFR1 and resistin. TNFR1 was positively correlated with resistin and leptin, as well as resistin with IL-8 and leptin.

**Conclusion:**

Increased levels of TNFR1, adipokines, chemokines and decrease of IL-4 play important role in the inflammatory process developed in T2DM and decreased renal function. We also suggest that TNFR1 is a strong predictor of renal dysfunction in patients with T2DM.

## Introduction

Type 2 diabetes mellitus (T2DM) is one of the most prevalent subtypes of diabetes mellitus (DM). It is a metabolic disorder resulting from the relative deficiency of insulin production and/or its action, which leads to increased serum glucose levels, which is considered the main cause of chronic kidney disease (CKD) [1, 2]. Hyperglycemia in T2DM is strongly associated with the development of macrovascular and microvascular complications, which may result in decreased renal function.[3] Studies suggest that low-grade inflammation, characterized by the production of cytokines, chemokines, and adipokines, is involved in the pathogenic processes that cause T2DM and its complications [4–7].

The imbalance between mediators triggers or enhances T2DM complications. Activation of the innate immune system alone induces hyperglycemia and insulin resistance. Thus, diabetes and inflammation are simultaneously involved, feeding a positive feedback loop [8].

Early identification of the risk of progressive loss of renal function in patients with T2DM might delay diabetes complications in these patients. Due to the lack of feasibility in measuring glomerular filtration rate, in clinical practice, the estimated glomerular filtration rate (eGFR) and albuminuria have been used as parameters to evaluate the renal function of patients with T2DM. An eGFR <60 mL/min/1.73 m^2^ might characterize decreased renal function [9, 10]. Plasma and urinary markers have recently shown that that early progressive renal decline, in the context of T2DM, has multiple causes [11].

Given the need to identify possible factors that contribute to low-grade inflammation and its complications, as reflected in the renal function of patients with T2DM, this study aimed to evaluate the serum concentrations of inflammatory mediators in patients with T2DM with or without renal alteration (RA), determined by the eGFR, and verify the correlation of these mediators to decreased renal function.

## Patients and methods

### Patients

Type 2 DM patients were recruited in the Endocrinology Outpatient Clinic of the Federal University of Triangulo Mineiro (UFTM), Uberaba, Minas Gerais, Brazil, between January to December of 2018. Healthy volunteers were recruited from the facilities of the Federal University of Triangulo Mineiro (UFTM), Uberaba, Minas Gerais, Brazil. Patients included in the study had T2DM diagnosis, age over 18 years-old and were in medical follow up in Endocrinology Outpatient Clinic of the UFTM. Healthy people aged above 18 years old and with normal renal function were also included for comparison. Pre-diabetic patients, T2DM patients aged under 18 years old and patients with T2DM and healthy people without sufficient data for eGFR calculation (age, race, serum creatinine and gender) were excluded from the study.

A total of 100 adult patients were recruited for this study, 76 of whom had T2DM (28 men and 48 women) and 24 were healthy volunteers (10 men and 14 women). The patients with T2DM were divided into two groups according to the eGFR (mL/min/1.73 m^2^) using the equation proposed by the Chronic Kidney Disease Epidemiology Collaboration study (CKD-EPI) [12]. These were the T2DM group without RA (n = 56, patients with T2DM with eGFR>60 mL/min/1.73 m^2^), with median age of 59.5 (18–84) years, and the T2DM group with RA (n = 20, patients with T2DM with eGFR <60 mL/min/1.73 m^2^), with median age of 75 (37–94) years. The control group consisted of 24 healthy patients without DM and with eGFR >60 mL/min/1.73 m^2^, with median age of 34 (22–58) years.

Because age differences between study participants are a limitation to be clarified, and age is considered a very important factor in the development of various entities, we used statistical tests to exclude the contribution of age difference in the studied sample. However, in the present study, it was possible to demonstrate that this parameter did not directly influence the evaluated markers. To demonstrate this result and eliminate any bias, we performed analyzes comparing elderly and non-elderly patients in the T2DM group without RA and elderly and non-elderly patients in the T2DM group with RA, as follows: T2DM group without RA: IL-4 (p = 0.3436; t = 0.9554); TNFR1 (p = 0.2640; t = 1,129); TNFR2 (p = 0.0476; t = 2,027); TNF-α (p = 0.5707; U = 276.5); IFN-γ (p = 0.0479; t = 2,024); IL-8 (p = 0.7313; U = 288.5); Eotaxin (p = 0.1895; U = 236); MIP-1α (p = 0.4993; U = 270.5); MIP-1β (p = 0.6570; U = 283); Adiponectin (p = 0.3885; U = 260); Resistin (p = 0.2334; U = 242.5) and Leptin (p = 0.4273; U = 264). T2DM group with RA: IL-4 (p = 0.6508; t = 0.4603); TNFR1 (p = 0.3352; U = 21); TNFR2 (p = 0.3352; U = 21); TNF-α (p = 0.3622; U = 22); IFN-γ (p = 0.8733; U = 30); IL-8 (p = 0.2054; U = 18); Eotaxin (p = 0.2114; U = 18); MIP-1α (p = 0.2799; U = 20); MIP-1β (p = 0.7505; U = 28); Adiponectin (p> 0.9999; U = 32); Resistin (p = 0.6993; t = 0.3926) and Leptin (p = 0.6167; U = 26). Given these results, it was possible to keep all patients in the study.

Clinical and laboratory data of the patients in the study were obtained from the information in the follow-up medical records of the patients with T2DM and the results of routine blood tests previously acquired from the volunteers.

The study was conducted in the laboratories of General Pathology Department and Immunology Department of the Federal University of Triângulo Mineiro (UFTM), Uberaba, Minas Gerais, Brazil. This study was approved by the Research Ethics Committee of the Federal University of Triângulo Mineiro under opinion number 3,001,006. All samples were archived and identified by codes with letters and numbers to ensure that individuals were anonymized. All patients and volunteers who were invited to participate in the study signed the informed consent form, after clarification.

## Methods

Patients with T2DM were approached at the time of routine clinical consultation, individually, at the doctor’s office. Healthy people recruited were referred to a reserved room in the General Pathology Department of UFTM. They were instructed about the research and those who agreed to participate, signed the consent form and had the biological sample collected. The biological sample was collected in a reserved and appropriate blood collection room, where general data of the participants were also recorded.

The sample was collected in a sterile tube, containing a separating gel, and centrifuged, after 30 min of rest, at 3,000 rpm, at 4°C, for 15 min to obtain the serum. The serum sample was stored at -80°C until analysis.

The serum cytokines were quantified using the Multiplex immunoassay—MAGPIX^™^ System (Lot #5028196) following the manufacturer’s instructions, in which the following mediators were detected: interleukin-4 (IL-4), interferon-γ (IFN-γ), tumor necrosis factor-α (TNF-α), IL-8 (CXCL8), eotaxin, macrophage inflammatory protein-1α (MIP-1α), and MIP-1β.

The adipokines (adiponectin, resistin, and leptin), tumor necrosis factor receptor-1 (TNFR1), and TNFR2 were measured by the quantitative sandwich enzyme-linked immunosorbent assay (ELISA) method using R&D Systems^®^ antibody pairs, following the manufacturer’s instructions: Human Adiponectin (Catalog DY1065), Human Resistin (Catalog DY1359), Human Leptin (Catalog DY398), Human TNF RI (Catalog DY225), and Human TNF RII (Catalog DY726).

### Statistical analysis

In the statistical analysis, an electronic spreadsheet (Microsoft Excel) was elaborated, and the data were analyzed using the GraphPad Prism software, version 7.0 (GraphPad Software, USA). The variables were tested for normality using the Kolmogorov-Smirnov test. For a non-normal distribution, we used the Mann-Whitney U test in the comparison between the two groups and the Kruskal-Wallis H test, followed by the Dunn’s post hoc test, among three or more groups. The proportions were compared using the chi-square test (χ^2^) or Fisher’s exact test. We used the Pearson’s r test to correlate parametric variables and the Spearman’s test (rS) for nonparametric variables. Differences were considered statistically significant when p *<* 0.05.

## Results

### Clinical and laboratory characteristics of the participants

A total of 100 patients were selected for the study and classified into three groups: the control group with 24 patients (24%), T2DM group without RA with 56 (56%) patients, and T2DM group with RA with 20 (20%) patients. According to the general characteristics of the groups, there was a predominance of women in the three groups, with 14 (58.3%) in the control group, 36 (64.3%) in the T2DM group without RA, and 12 (60%) in the T2DM group with RA. The patients were mainly Caucasian, with 20 (83.3%) in the control group, 45 (80.4%) in the T2DM group without RA, and 16 (80%) in the T2DM group with RA. Most patients with T2DM had hypertension, with 36 (64.3%) in the T2DM group without RA and 18 (90%) in the T2DM group with RA, whereas there was no patient with hypertension in the control group. The body mass index (BMI) was higher in the T2DM groups than in the control group. Patients with T2DM with RA had longer DM duration compared to patients with T2DM without RA. Moreover, most patients in both groups reported the use of insulin to control diabetes, with 38 (67.8%) in the T2DM group without RA and 17 (85%) in the T2DM group with RA.

Regarding the laboratory data, as expected, fasting serum glucose and glycated hemoglobin levels were higher in patients with T2DM. Serum urea and creatinine levels were higher in the T2DM group with RA than in other groups. Serum total cholesterol, high-density lipoprotein cholesterol, and triglyceride levels were similar among groups. However, serum low-density lipoprotein cholesterol levels were higher in the control group than in other groups. Regarding the habit of drinking alcohol, most 14 (58.3%) patients in the control group reported social use, while 30 (53.6%) patients in T2DM group without RA and 12 (60%) patients in T2DM group with RA reported not to consume alcohol. Most patients in all groups were non-smokers. Regarding physical activities, half of the patients both in control and T2DM with RA groups did not practice physical activities as well as 35 (62.5%) patients of T2DM group without RA. The clinical and laboratory characteristics of the patients are detailed in Table 1.

**Table 1:** Clinical and laboratory data of diabetic and control groups.

	Control (n = 24)	T2DM without RA (n = 56)	T2DM with RA (n = 20)
**Age (years)**			
Median (Min-Max)	34 (22–58)	59.5 (18–84)	74 (37–94)
**Gender n (%)**			
Male	10 (41.7%)	20 (35.7%)	08 (40%)
Female	14 (58.3%)	36 (64.3%)	12 (60%)
**Color n (%)**			
White	20 (83.3%)	45 (80.4%)	16 (80%)
Not white	04 (16.7%)	11 (19.7%)	04 (20%)
**SAH n (%)**			
Yes		36 (64.3%)	18 (90%)
No	24 (100%)	20 (35.7%)	02 (10%)
**BMI (kg/m**^**2**^**)**			
Median (Min-Max)	24.34 (19.6–30.4)	27.19 (18.17–42.1)	26.5 (18.4–36.8)
**Course DM (years)**			
Median (Min-Max)		11.5 (0.4–30)	17.5 (0.5–40)
**Insulin n (%)**			
Yes		38 (67.8%)	17 (85%)
No		18 (32.2%)	03 (15%)
**eGFR (mL/min/1.73m**^**2**^**)**			
Mean ± SD	93.81 ± 18.14	89.55 ± 21.17	40.72 ± 16.95
**Fasting glucose (mg/dL)**			
Median (Min-Max)	86.9 (69–101)	169.9 (94.9–596.9)	143.3 (53.7–366.7)
**HgA1c (%)**			
Median (Min-Max)	4.8 (4.5–5.7)	8.6 (5.9–15.1)	7.6 (5.3–10.9)
**Urea (mg/dL)**			
Median (Min-Max)	32 (23.3–40)	29 (18.4–61.7)	56.5 (34.1–191.9)
**Creatinine (mg/dL)**			
Median (Min-Max)	0.87 (0.6–1.4)	0.82 (0.41–1.3)	1.27 (0.95–10.61)
**TC (mg/dL)**			
Median (Min-Max)	183.1 (148–282)	162.9 (93.3–275.3)	168(104.3–265.3)
**HDL (mg/dL)**			
Median (Min-Max)	53 (27–76)	52 (26–97)	50 (32–101)
**LDL (mg/dL)**			
Median (Min-Max)	98 (74–138)	67.6 (24–192.3)	78.8 (48.7–186.2)
**TG (mg/dL)**			
Median (Min-Max)	97 (46–235)	125.5 (28–558)	151 (32–323)
**Alcohol drinking habit n (%)**			
Yes (socially)	14 (58.3%)	9 (16.1%)	1 (5%)
No	9 (37.5%)	30 (53.6%)	12 (60%)
Past		5 (8.9%)	
NI	1 (4.2%)	12 (21.4%)	7 (35%)
**Smoking n (%)**			
Yes	1 (4.2%)	6 (10.7%)	
No	22 (91.6%)	32 (57.1%)	12 (60%)
Past		9 (16.1%)	2 (10%)
NI	1 (4.2%)	9 (16.1%)	6 (30%)
**Practicing exercise n (%)**			
Yes	11 (45.8%)	21 (37.5%)	10 (50%)
No	12 (50%)	35 (62.5%)	10 (50%)
NI	1 (4.2%)		

DM: Diabetes Mellitus. RA: Renal alteration. SAH: Systemic arterial hypertension. BMI: Body mass index. MG: Minas Gerais. eGFR: Estimated glomerular filtration rate. HgA1c: Glycated hemoglobin. TC: Total cholesterol. HDL: High density lipoprotein. LDL: Low density lipoprotein. TG: Triglycerides. NI: Not informed. SD: Standard deviation.

### Imbalance in serum cytokine production in patients with T2DM with RA

**Fig 1:**
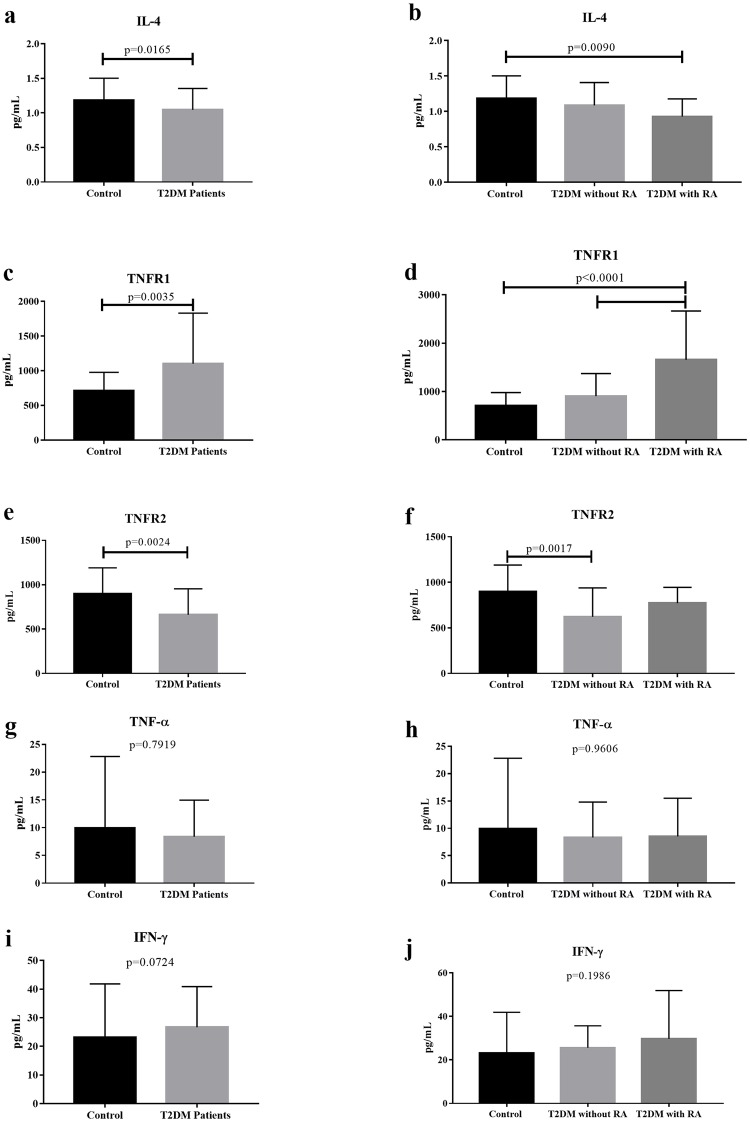
Serum cytokine concentrations in the T2DM group without and with RA and control group. (a) Serum IL-4 level in T2DM patients and controls and (b) T2DM patients without and with RA vs. controls. (c) Serum TNFR1 level in T2DM patients and controls and (d) T2DM patients without and with RA vs. controls. (e) Serum TNFR2 level in T2DM patients and controls and (f) T2DM patients without and with RA vs. controls. (g) Serum TNF-α in T2DM patients and controls and (h) T2DM patients without and with RA vs. controls. (i) Serum INF-γ level in T2DM patients and controls and (j) T2DM patients without and with RA vs. controls. The results were expressed as mean ± standard deviation. RA, renal alteration.

Inflammatory cytokines were analyzed in patients with T2DM to evaluate their production in the context of renal function. Patients with T2DM showed a significant decrease in serum IL-4 and TNFR2 levels (p = 0.0165, U = 617, and p = 0.0024, U = 541.5, respectively) and a significant increase in TNFR1 level compared to controls (p = 0.0035; U = 554.5). However, there was no significant difference in TNF-α level between the groups (p = 0.7919; U = 879), and there was only a tendency of increased IFN-γ level in patients with T2DM (p = 0.0724; U = 690). Comparing the groups based on RA, the T2DM group with RA had decreased IL-4 levels compared to the control group (p = 0.0090; H = 9.413, Dunn’s post hoc test) and increased TNFR1 levels when compared to the T2DM group without RA and control group (p<0.0001; H = 20.58, Dunn’s post hoc test). Patients without RA showed a decrease in TNFR2 level compared to the control group (p = 0.0017; H = 12.76, Dunn’s post hoc test), and there was no significant difference in TNF-α (p = 0.9606; H = 0.0803, Dunn’s post hoc test) and IFN-γ levels (p = 0.1986; H = 3.233 Dunn’s post hoc test) between the groups (Fig 1).

### Increased serum adipokine production in patients with T2DM with RA

**Fig 2:**
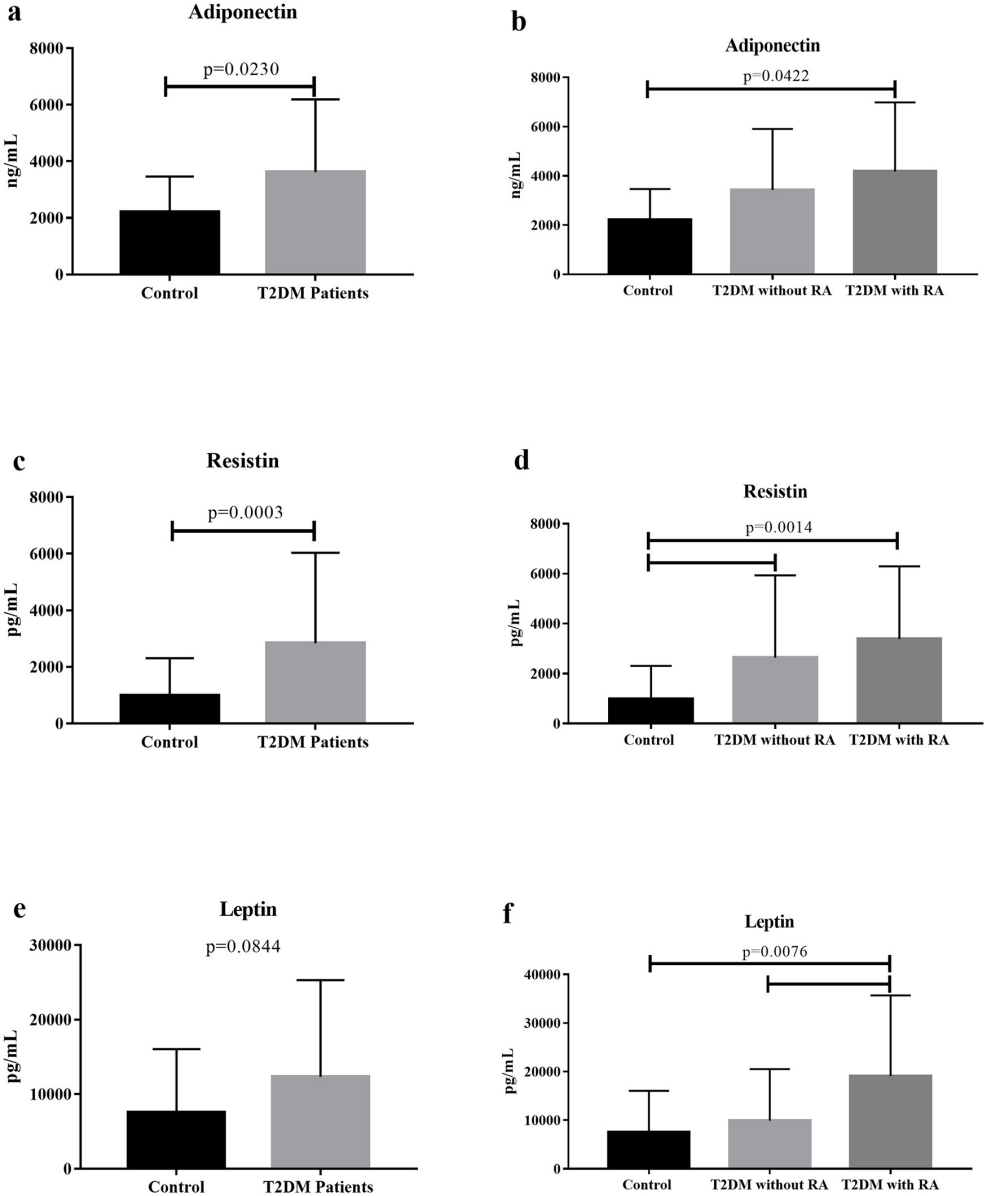
Serum adipokine levels in T2DM group without and with RA and control group. (a) Serum adiponectin levels in T2DM patients and controls and (b) T2DM patients without and with RA vs. controls. (c) Serum resistin level in T2DM patients and controls and (d) T2DM patients without and with RA vs. controls. (e) Serum leptin level in T2DM patients and controls and (f) T2DM patients without and with RA vs. controls. The results were expressed as mean ± standard deviation. RA, renal alteration.

Observing the imbalance in serum cytokine production in patients with T2DM with RA and considering T2DM as a low-grade chronic inflammatory process, we analyzed adipokine production in these patients. There was a significant increase in serum adiponectin and resistin levels in patients with T2DM compared to the control group (p = 0.0230, U = 631.5, and p = 0.0003, U = 478.5, respectively). Moreover, there was a tendency for increased leptin levels in patients with T2DM compared to that in the control group (p = 0.0844; U = 698). Comparing the groups based on RA, there was an increase in adiponectin levels in the patients with T2DM with RA compared to the control group (p = 0.0422; H = 6.329, Dunn’s post hoc test). Regardless of RA, patients with T2DM showed an increase in resistin levels compared to the control group (p = 0.0014; H = 13.12, Dunn’s post hoc test). However, the serum leptin levels were significantly higher in the T2DM group with RA compared to those in the T2DM group without RA and control group (p = 0.0076; H = 9.759, Dunn’s post hoc test) (Fig 2).

### Increased serum chemokine production in patients with T2DM with RA

Observing the increase in adipokine production associated with the imbalance in cytokine production in patients with T2DM with RA, we analyzed the chemokine production in these patients. There was a significant increase in serum IL-8 (p<0.0001; U = 322), eotaxin (p = 0.0330; U = 648.5), MIP-1α (p = 0.0011; U = 541), and MIP-1β (p = 0.0380; U = 655.5) levels in T2DM patients compared to those in the control group.

**Fig 3:**
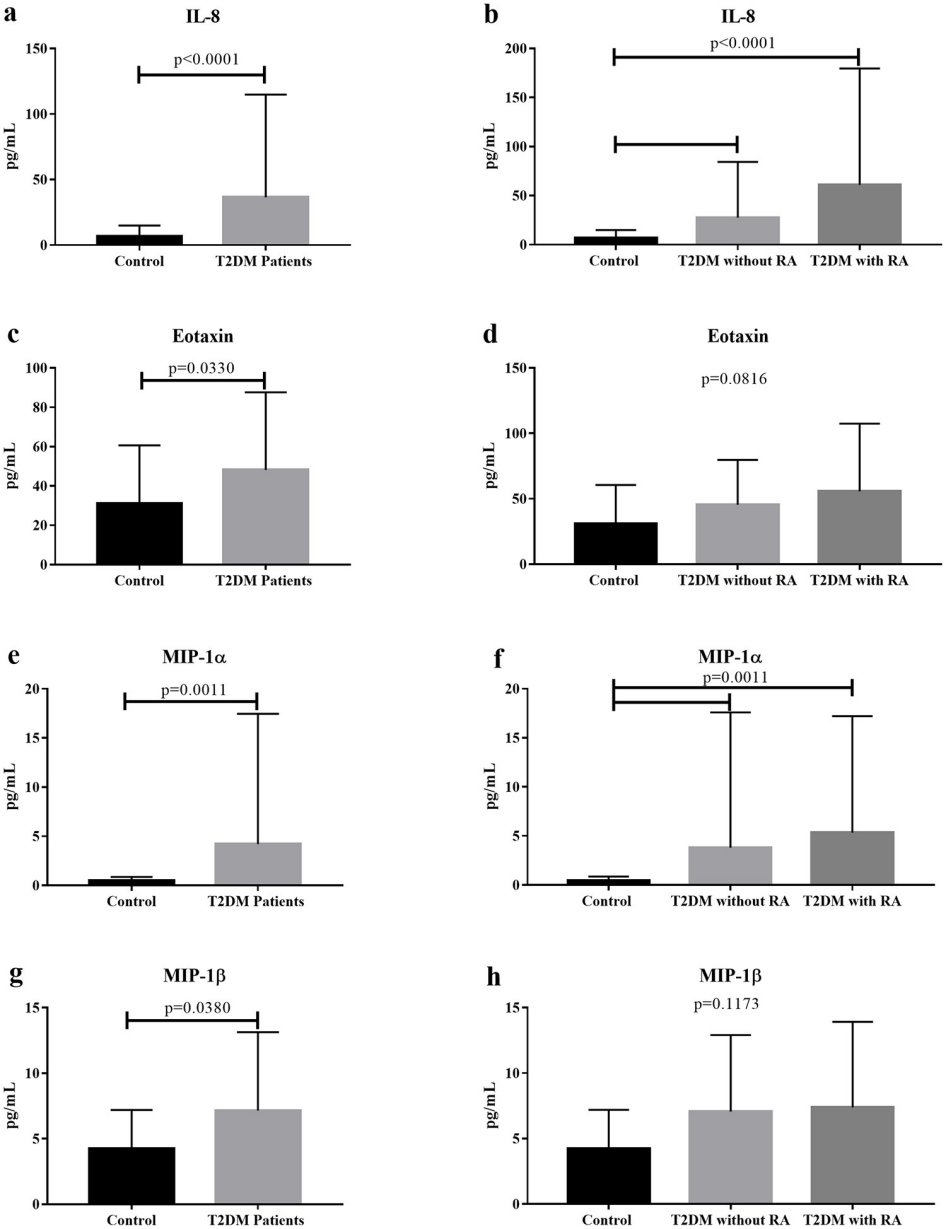
Serum chemokine levels in the T2DM group without and with RA and control group. (a) Serum IL-8 level in T2DM patients and controls and (b) T2DM patients without and with RA vs. controls. (c) Serum eotaxin level in T2DM patients and controls and (d) T2DM patients without and with RA vs. controls. (e) Serum MIP-1α level in T2DM patients and controls and (f) T2DM patients without and with RA vs. controls. (g) Serum MIP-1β level in T2DM patients and controls and (h) T2DM patients without and with RA vs. controls. The results were expressed as mean ± standard deviation. RA, renal alteration.

Comparing the groups based on RA, it was found that, regardless of RA, serum IL-8 levels remain significantly elevated in the T2DM group without RA and T2DM group with RA compared to that in the control group (p<0.0001; H = 22.8, Dunn’s post hoc test). The same mechanism was observed with regard to the MIP-1α level (p = 0.0011; H = 13.61, Dunn’s post hoc test). There was no significant difference in eotaxin (p = 0.0816; H = 5.011, Dunn’s post hoc test) and MIP-1β levels (p = 0.1173; H = 4.286, Dunn’s post hoc test) between the groups. However, it is possible to observe that both eotaxin and MIP-1β tend to behave similarly to IL-8 and MIP-1α (Fig 3).

### Correlation between eGFR and cytokines/ chemokines/ adipokines in patients with T2DM with RA

**Fig 4:**
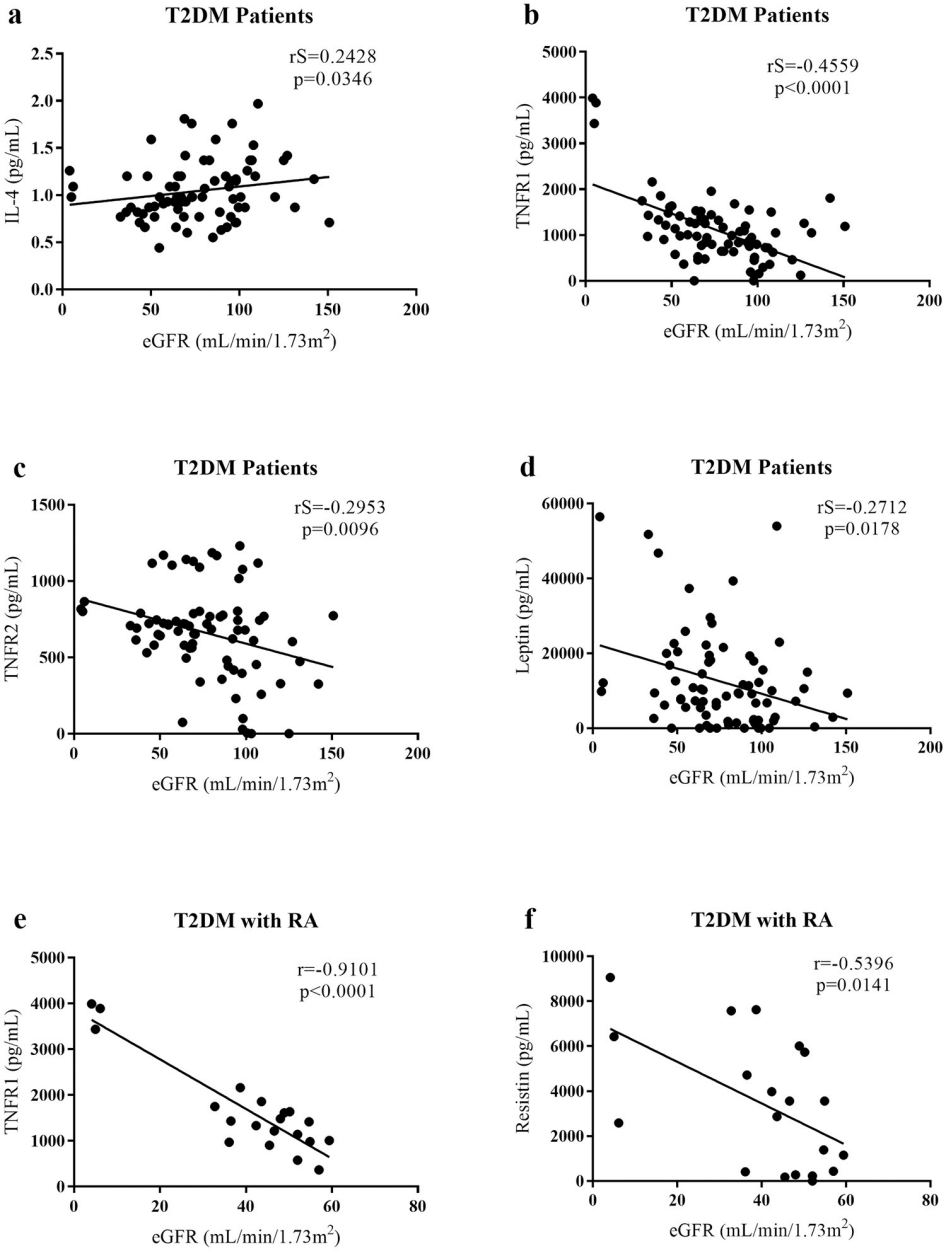
Correlations between cytokine serum levels and estimated glomerular filtration rate (eGFR) in T2DM patients and T2DM with RA. (a) Positive and significant correlation between IL-4 level and eGFR in T2DM patients. (b) Negative and significant correlation, in T2DM patients, between TNFR1 level and eGFR, (c) between TNFR2 level and eGFR and (d) between leptin level and eGFR. (e) Negative and significant correlation, in T2DM with RA group, between TNFR1 level and eGFR and (f) between resistin level and eGFR. RA, renal alteration.

To evaluate the correlation of mediators in patients with T2DM with RA, correlations between eGFR and cytokines/chemokines/adipokines were analyzed. Patients with T2DM had a positive and significant correlation between eGFR and IL-4 (p = 0.0346; rS = 0.2428) and a negative and significant correlation between eGFR and TNFR1 (p<0.0001; rS = -0.4559), TNFR2 (p = 0.0096; rS = -0.2956), and leptin (p = 0.0178; rS = -0.2712). In the T2DM group with RA, eGFR was negatively and significantly correlated with TNFR1 (p<0.0001; r = -0.9101) and resistin (p = 0.0141; r = -0.5396, Fig 4).

**Fig 5:**
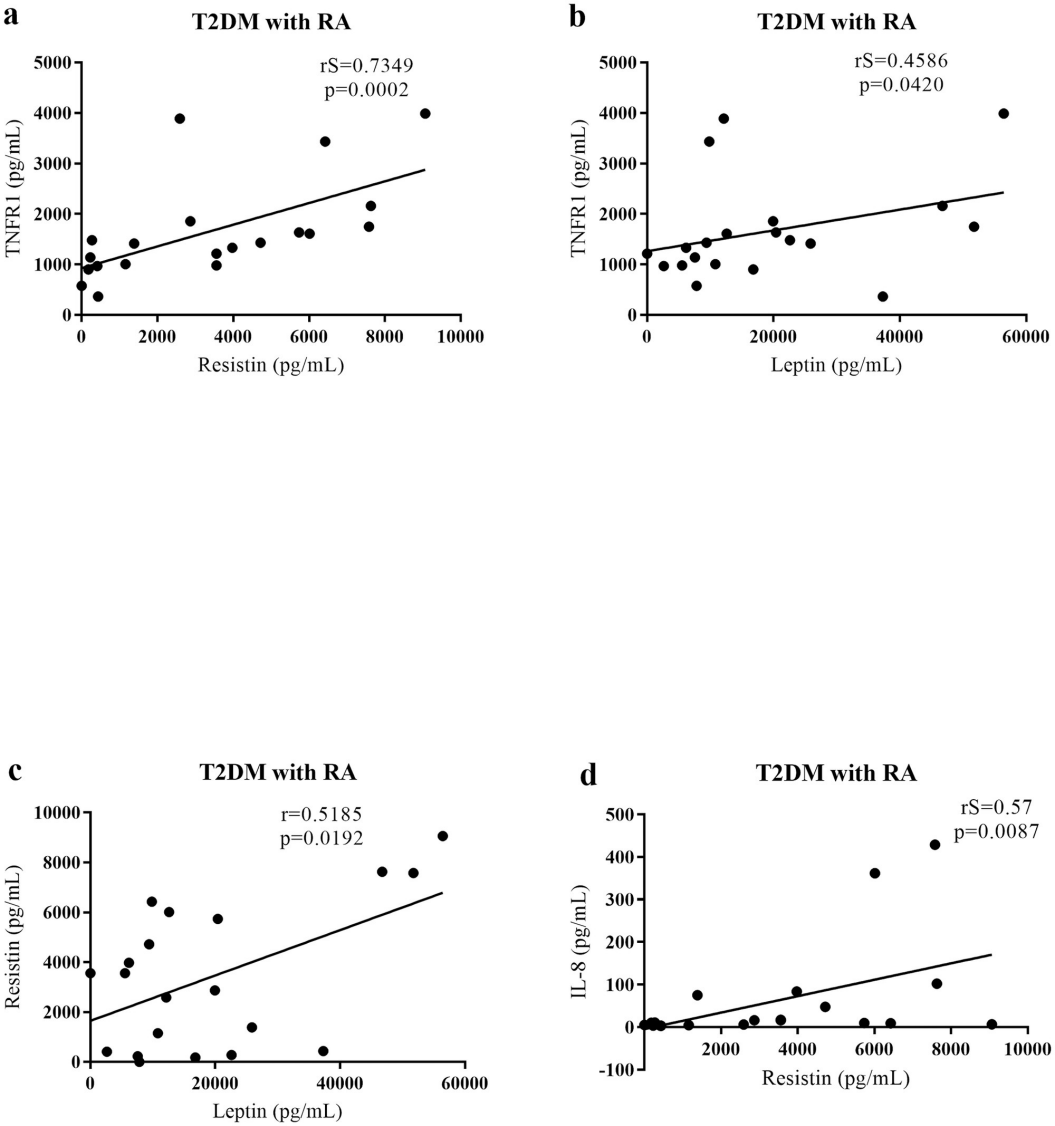
Correlations between serum cytokine and adipokine levels in patients with T2DM with RA. (a) Positive and significant correlation between TNFR1 and resistin levels, (b) between TNFR1 and leptin levels, (c) between resistin and leptin levels, (d) between resistin and IL-8 levels in patients with T2DM with RA. RA, renal alteration.

Patients with T2DM with RA showed a positive and significant correlation between TNFR1 and resistin (p = 0.0002; rS = 0.7349) and leptin (p = 0.0420; rS = 0.4586). A positive and significant correlation was also observed between resistin and leptin (p = 0.0192; r = 0.5185) and between resistin and IL-8 (p = 0.0087; rS = 0.57, Fig 5).

## Discussion

Although hyperglycemia is considered the main triggering factor of Diabetic Nephropathy (DN), low-grade chronic inflammation is one of the triggering factors of kidney injury in patients with T2DM [13–15]. Several studies are being conducted to determine the actual role of inflammatory cytokines in the development and progression of diabetic kidney disease [16–18]. In this context, this study analyzed the serum cytokine/chemokine/adipokine levels in patients with T2DM with or without RA as determined by eGFR in relation to those in healthy patients, to investigate the association of these inflammatory mediators with decreased renal function.

In our study, patients with T2DM had decreased serum IL-4 levels compared to the control group. A decreased IL-4 level was also found in patients with T2DM with RA compared to that in the control group, indicating that not only diabetes but also RA characterized by eGFR <60 mL/min/1.73 m^2^ is associated with decreased IL-4 level. IL-4 is a Th2 profile anti-inflammatory cytokine that acts to reduce the secretion of proinflammatory cytokines by activated macrophages and stimulates the production of a number of anti-inflammatory molecules, such as IL-1ra [19], IL-1R2 [20], and soluble TNF receptors [21]. Patients with DN [22], as well as those with T2DM [23], show decreased serum IL-4 levels. Results of experimental studies with db/db mice suggested that suppression of the inflammatory process by anti-inflammatory cytokines is impaired in T2DM [24, 25]. The decrease in serum IL-4 levels compromises its action in reducing the effects of IL-1 and IL-8 [26, 27], determining a factor of worse evolution of T2DM. Thus, the decrease in IL-4 level may be associated with the development of inflammatory process complications. This culminates in impairing the renal function of these patients, since serum IL-4 level decreases as eGFR decreases, as shown by the positive and significant correlation between IL-4 level and eGFR. Possibly, the decrease in serum IL-4 levels in the patients of this study could be due to the increase in cytokine, adipokine, and chemokine levels.

In this study, the results of patients with T2DM were different with respect to serum TNFR levels as TNFR1 level increased and TNFR2 level decreased compared to those in the control group. One of the main findings of our study was that, exclusively, the increase in TNFR1 level distinguished the patients with T2DM with RA from those with T2DM without RA and healthy volunteers. This fact was confirmed by the negative and significant correlation between eGFR and TNFR1 in patients with T2DM and even stronger correlation in patients with T2DM with RA. Thus, this result shows that TNFR1 can predict a decrease in renal function in patients with T2DM. Although TNFR2 is decreased in patients with T2DM without RA compared to those in the control group, it was noted that its serum level might increase in patients with T2DM with RA, which was strengthened by the fact that eGFR correlates negatively with TNFR2 level. Nevertheless, despite the relevance of the results found with their receptors, we did not observe any significant difference between the groups in relation to TNF-α.

TNF-α is a pleiotropic cytokine that plays an important role in the mediation of inflammatory processes. It is a transmembrane homotrimeric protein, which is produced by many cells, including fat, endothelial cells, and leukocytes. In plasma, TNF-α appears free or bound to the circulating TNFR1 and TNFR2 [28]. In a 12-year follow-up study conducted in patients with T2DM, it was observed that, of all markers analyzed, only TNFR1 and TNFR2 were associated with the risk of end-stage renal disease. A stronger association was found with TNFR1, suggesting that high serum levels of this receptor can predict the progression of T2DM to CKD [29]. Other studies have shown that elevated plasma TNFR1 levels are associated with decreased eGFR in patients with T2DM [30, 31], which corroborates our findings. In contrast, a recent study showed that patients with T2DM had increased plasma levels of not only TNFR1 but also TNF-α and TNFR2 compared to the control group, which differs from our findings. However, similar to our results, TNFR1 and TNFR2 were strongly associated with kidney injury [32]. It is still unclear why serum TNFR levels are more closely associated with eGFR. One possible explanation is that, because TNFR levels are at least 100 times greater than TNF-α levels, circulating TNFRs play an important role in the progression of diabetic kidney disease, regardless of the TNF-α levels [33].

In association with the cytokine findings, our study found an increase in serum adipokine levels in patients with T2DM. Patients with T2DM with RA showed a significant increase in adiponectin level. Regardless of RA, patients with T2DM also showed an increase in resistin level. However, serum resistin levels tend to increase as eGFR decreases, which was confirmed by the negative and significant correlation found between eGFR and resistin level. Additionally, another important finding of our study was in relation to the serum leptin levels, which established its importance in distinguishing patients with T2DM with RA, also showing a negative and significant correlation with eGFR. In light of these findings, our results demonstrate that an increase in adipokine levels is related to a decrease in renal function in patients with T2DM.

Adiponectin is an adipokine secreted exclusively by human adipocytes [34]. It has beneficial effects on insulin resistance and anti-inflammatory [35] and anti-oxidative properties [36]. It is suggested that the anti-inflammatory action of adiponectin is due to the inhibition of proinflammatory cytokine production, such as IL-6 and TNF-α, by macrophages and/or reduction of their phagocytic action [37]. We observed a significant increase in adiponectin level in our patients. Perhaps, this factor has contributed in the serum levels of proinflammatory cytokines, such as TNF-α and IFN-γ. Although some studies reported that patients with T2DM have lower circulating quantities of adiponectin than those without T2DM [38, 39], other studies have shown that, under various kidney disease conditions [40, 41] and in patients with T2DM with CKD [42, 43] the serum adiponectin levels are increased, which corroborates our findings. Another study evaluating more than 1,200 patients with T2DM showed an inverse correlation between serum adiponectin levels and eGFR [44]. The correlation of adiponectin and CKD is still controversial. It is suggested that, in individuals with kidney dysfunction, increased adiponectin levels represent not only a decrease in renal excretion but also a temporary homeostatic mechanism in an attempt to reduce renal damage through anti-inflammatory and anti-oxidative mechanisms [45, 46].

Resistin is a protein secreted mainly by macrophages and monocytes in humans and has proinflammatory effects [47, 48]. The association between serum resistin levels and CKD in diabetes is also unclear. It was recently observed that patients with microalbuminuria and T2DM with eGFR <60 mL/min/1.73 m^2^ showed a significant increase in serum resistin levels compared to patients with T2DM with normal renal function. Additionally, serum resistin levels were correlated negatively with eGFR and positively with C-reactive protein level. Thus, the main determinants of resistin levels in patients with T2DM are renal function level and inflammation [7]. Axelsson et al. demonstrated that high resistin levels in patients with T2DM with CKD were associated with decreased eGFR and inflammation [49]. A prospective cohort study showed that high resistin and TNFR2 levels are related to a higher risk of decline in renal function [50]. Moreover, an increase in resistin levels was observed in the early stages of CKD [51]. This means that even in mild renal function, there is already an increase in resistin level, which corroborates our findings. In agreement, other investigations suggest that resistin might promote endothelial dysfunction by enhancing the oxidative stress, an effect that would eventually culminate in glomerular dysfunction [52, 53] and that the adverse effects of resistin could be attributed to its ability to stimulate proinflammatory cytokine production [47, 54].

Another adipokine with proinflammatory effects, which promotes the synthesis of other inflammatory cytokines, is leptin. It is involved in the control of food intake, leading to appetite suppression. Patients with obesity have hyperleptinemia due to the development of leptin resistance [55]. High leptin levels are associated with insulin resistance and development of T2DM [56]. It has been shown that an increase in serum leptin levels are related to a decline in eGFR, and this association has been described to be stronger in women [57] and patients with CKD [58]. Both the decrease and increase in leptin levels are risk factors for the decline in renal function in patients with T2DM [59]. Our results showed that patients with T2DM with decreased renal function had increased serum leptin levels. In addition to a decreased renal excretion due to renal dysfunction, unfavorable actions of leptin, such as the activation of the sympathetic nervous system, rather than causing beneficial effects, may affect the renal function decline in patients with hyperleptinemia. This can be then further compromised, due to the leptin resistance found in these patients [59].

Among the chemokines analyzed, we observed that patients with T2DM had a significant increase in serum IL-8, eotaxin, MIP-1α, and MIP-1β levels compared to the control group. One study evaluated urinary cytokine levels in patients with T2DM with normo- and microalbuminuria and found a significant increase in urinary IL-8, IP-10, MCP-1, G-CSF, eotaxin, RANTES, and TNF-α levels in patients with microalbuminuria compared to patients with normoalbuminuria. Patients with microalbuminuria had a significant increase in GM-CSF, MIP-1α, and MIP-1β levels compared to the control group. These results indicated that determination of the urine cytokine level might be useful in the diagnosis and early treatment of diabetic nephropathy [60].

Our results showed that the increase in serum IL-8 levels in patients with T2DM is independent of the presence of RA, although its increase, accompanied by decrease in eGFR, is noticeable. IL-8 (CXCL8) was the first chemokine to be discovered and has a predominantly chemoattractant effect on neutrophils [61, 62]. It enhances the expression of adhesion molecules by endothelial cells and antagonizes IgE production stimulated by IL-4 [63]. It is produced mainly by monocytes/macrophages and, to a lesser extent, by fibroblasts, endothelial cells, keratinocytes, hepatocytes, melanocytes, and chondrocytes. IL-1, TNF-α, and IFN-γ are its main stimulators [64]. In the kidneys, podocytes and endothelial cells of interstitial vessels are the main sources of IL-8, while tubular epithelial cells express small amounts of this cytokine. In inflammatory kidney diseases, the IL-8 expression increases fivefold compared to those in normal structures. It increases the level of endothelial cells near the inflammatory site, facilitates the recruitment and crossing of leukocytes through the endothelium, and alters the expression of adhesion molecules [65]. Urinary IL-8 levels have been observed to be elevated in the early stages of diabetic nephropathy in patients with T2DM [66]. Another study that evaluated the association between urinary cytokine levels and decreased eGFR in patients with T2DM with DN found that increased urinary levels of IL-6, IL-8, TNF-α, and TFG-β were predictors of a faster decline in renal function, indicating the clinical utility of these levels in stratifying the risk of renal disease progression [67]. In patients with T2DM, IL-8 was negatively associated with eGFR and positively associated with BMI [68]. These studies reveal that there is an association between increased IL-8 level and decreased eGFR in patients with T2DM. Our results showed that the increase in serum IL-8 level anticipated a decrease in renal function in patients with T2DM. A possible explanation is that the hyperglycemic environment itself promotes increased serum levels of this chemoattractant cytokine. These contribute to the onset and progression of the inflammatory process, from recruitment, especially of neutrophils, to vascular changes, such as increased permeability that favors the arrival of new inflammatory cells to the inflammatory site, which results in renal function impairment [69].

Similar to that found in relation to serum IL-8 levels, in our study, the patients with T2DM also showed an increase in serum MIP-1α and MIP-1β levels compared to the control group. However, only MIP-1α level showed a significant difference in the evaluation of T2DM group without RA and T2DM group with RA in relation to the control group. Thus, similar to the IL-8 level, the increase in serum MIP-1α levels in patients with T2DM is independent of the presence of RA. However, its increase also seems to accompany the decrease in eGFR. MIP-1α (CCL3) and MIP-1β (CCL4) belong to the CC subfamily of chemokines and induce the expression of adhesion and costimulatory molecules on the surface of T cells, NK cells, macrophages, and monocytes. These chemokines not only mediate the chemotaxis of these cells but also promote the secretion of proinflammatory cytokines [70]. One study evaluated the serum levels of inflammatory cytokines in 64 patients with T2DM with CKD, and it was observed that patients with eGFR of 30–59 mL/min/1.73 m^2^ had increased serum MIP-1α levels. This was associated with the decline in eGFR and also correlated positively with urinary albumin excretion [71]. Patients with T2DM with diagnosis of DN showed an increase in serum MIP-1β levels in CKD stages 1–2 [72]. Thus, corroborating these studies, our results suggest that increased serum MIP-1α and MIP-1β levels may anticipate the decline in renal function of patients with T2DM.

In this study, patients with T2DM showed an increase in serum eotaxin levels compared to the control group. However, there was no difference between patients with T2DM with and those without RA. Although there was no such difference, a trend of increased serum eotaxin levels in these patients was noted. Eotaxin is a CC chemokine that acts on chemotaxis, mainly of eosinophils. It is secreted by endothelial cells, macrophages, fibroblasts, and smooth muscle cells [73]. In 2015, a study conducted in African American patients with type 1 diabetes was the first to report that increased plasma eotaxin levels are an independent predictor of renal failure [74]. A prolonged hyperglycemia process increases the excretion of urinary eotaxin and other inflammatory mediators [75]. Increased urinary eotaxin levels were found in patients with microalbuminuria and T2DM compared to patients with normoalbuminuria and controls [60]. In addition to angiogenic properties [76] and contributing to renal interstitial eosinophilia [77], studies have shown that an increase in serum eotaxin levels in patients with T2DM could play an important role in the process of atherosclerosis that developed in patients with T2DM [78] and chronic renal disease [72]. Increased serum eotaxin levels were also observed in obese mice and humans [79]. Thus, it is possible to associate the increase in serum and urinary eotaxin levels with the development of T2DM complications and renal function impairment, as probably related to obesity that may affect patients with T2DM and favor the low-grade chronic inflammatory process [72, 79].

Low-grade chronic inflammation promoted by T2DM is associated with macrophage infiltration in the kidney. Monocytes/macrophages and neutrophils are considered primordial cells that drive inflammation and concomitant production of proinflammatory cytokines in vivo [80, 81]. Increased infiltration of activated monocytes, macrophages, and T lymphocytes are described in the kidneys of patients with T2DM with DN [82, 83] reinforcing the hypothesis that T2DM is a disease of the innate immune system [84, 85]. Thus, our study demonstrates that patients with T2DM with RA show increased stimulation for the recruitment of innate immune cells, through an increase in the serum levels of pro-inflammatory chemokines, stimulated by the increase in adipokines and TNFR1, with consequent decrease in IL-4, favoring the inflammatory process. Hence, this immune mechanism could be associated with and plays an important role in promoting the decline in renal function in patients with T2DM. Therefore, our study highlights the importance of this screening for increased serum TNFR1, adipokine, and chemokine levels and decreased serum IL-4 level in patients with T2DM to identify individuals at risk of progressive loss of renal function.

We demonstrated that TNFR1 correlated positively with resistin and leptin in patients with T2DM with RA, showing its contribution to the increase of these adipokines in conditions of decreased renal function. The relationship between these molecules appears to be complex, and there are still several points related to their signaling that need to be better understood. Although studies such as Fasshauer *et al*., 2001, have shown a negative effect of resistin on TNF-α production in an adipocyte cell line [86], several studies have shown that TNF-α signaling via TNFR1 is a strong stimulus for resistin and leptin production [47, 87–90]. Resistin also acts by stimulating proinflammatory cytokines such as TNF-α and IL-12 [91, 92], and may be the link between inflammation and insulin resistance in an inflammatory environment [93, 94]. Moreover, the relationship between adipokines, TNF-α and their receptors has been reported in other studies on diseases with an important inflammatory component, such as Lupus [95], Inflammatory Bowel Disease [96], rheumatoid arthritis [47, 97, 98], atherosclerosis [99, 100] and chronic kidney disease in the absence of Diabetes Mellitus [101, 102].

The real role of leptin and resistin as risk indicators for kidney injury has yet to be further clarified. Studies have reported that leptin is inversely related to glomerular filtration rate [103, 104] and positively associated with chronic kidney disease [58]. Leptin is metabolized mainly by renal proximal tubular cells and decreased glomerular filtration rate may result in decreased leptin clearance and therefore higher serum leptin levels. High serum leptin levels were observed in the early stages of kidney disease in T2DM patients, demonstrating that leptin degradation is already impaired in the early stages of nephropathy [105].

Studies have also reported increased serum resistin inversely associated with estimated glomerular filtration rate in T2DM patients [49, 106]. Individuals with renal dysfunction have accumulated serum resistin levels, which is possibly due to reduced renal clearance. However, resistin levels are significantly increased even in individuals with eGFR between 60–89 mL/min/ 1.73 m2, where polypeptides would be filtered almost normally [51]. However, serum resistin levels are still significantly higher in patients with mild renal dysfunction than in those with eGFR> 90 mL/min/ 1.73 m^2^.

Thus, it is hypothesized that resistin plays an important role in decreasing renal function [51], possibly through its proinflammatory effect that may be detrimental to renal function [106]. Mills and colleagues reported that leptin and resistin are significantly associated with the risk and severity of chronic kidney disease [104]. Thus, we can suggest that possibly increased levels of resistin and leptin associated with increased levels of TNFR1 may indicate the risk of renal injury in T2DM patients.

Furthermore, under this same condition, resistin was shown to be correlated positively to leptin and IL-8. Thus, adipokines, especially resistin and leptin, TNFR1, and IL-8, exert similar behaviors in patients with T2DM with decreased renal function in their inflammatory process.

## Conclusions

Our study showed that serum TNFR1, IL-4, adipokines, and chemokines play an important role in the inflammatory process in T2DM and decreased renal function. Moreover, our data indicate that TNFR1 is a strong predictor of renal dysfunction in patients with T2DM.
